# Zinc in Keratinocytes and Langerhans Cells: Relevance to the Epidermal Homeostasis

**DOI:** 10.1155/2018/5404093

**Published:** 2018-12-09

**Authors:** Youichi Ogawa, Manao Kinoshita, Shinji Shimada, Tatsuyoshi Kawamura

**Affiliations:** Department of Dermatology, Faculty of Medicine, University of Yamanashi, Yamanashi 409-3898, Japan

## Abstract

In the skin, the epidermis is continuously exposed to various kinds of external substances and stimuli. Therefore, epidermal barriers are crucial for providing protection, safeguarding health, and regulating water balance by maintaining skin homeostasis. Disruption of the epidermal barrier allows external substances and stimuli to invade or stimulate the epidermal cells, leading to the elicitation of skin inflammation. The major components of the epidermal barrier are the stratum corneum (SC) and tight junctions (TJs). The presence of zinc in the epidermis promotes epidermal homeostasis; hence, this study reviewed the role of zinc in the formation and function of the SC and TJs. Langerhans cells (LCs) are one of the antigen-presenting cells found in the epidermis. They form TJs with adjacent keratinocytes (KCs), capture external antigens, and induce antigen-specific immune reactions. Thus, the function of zinc in LCs was examined in this review. We also summarized the general knowledge of zinc and zinc transporters in the epidermis with updated findings.

## 1. Introduction

The epidermis is the outermost layer of the skin and is thus continuously exposed to various kinds of external substances and stimuli that can lead to potential harm. To counteract these risks and maintain homeostasis, the epidermis provides a barrier against the external environment. The importance of preserving epidermal homeostasis is evidenced by a reduction in the development of atopic dermatitis (AD) when moisturizer is applied to the skin during the neonatal period [1, 2]. Reports also suggest that skin barrier disruption leads not only to the development of AD but also to other allergic diseases such as asthma, food allergies, and allergic rhinitis [3, 4].

**Figure 1:**
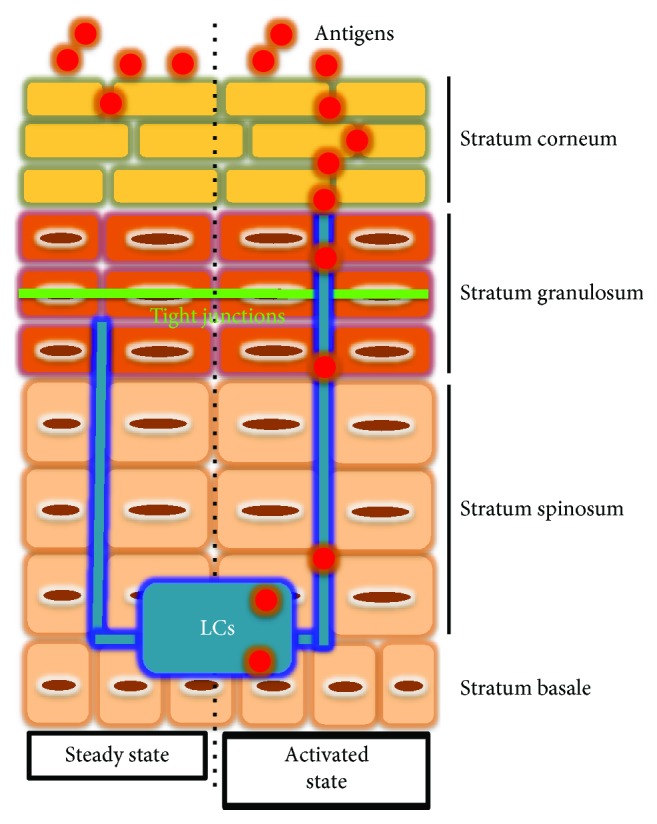
Structure of the epidermis. The epidermal KCs are categorized into four layers, namely, the stratum basale, stratum spinosum, stratum granulosum (SG), and stratum corneum (SC). A functional epidermal barrier depends on the existence of the SC and tight junctions (TJs) formed in the SG. LCs can link with KCs to form TJs. In steady state (left), LC dendrites lie beneath the TJs. Upon activation (right), LCs elongate their dendrites to penetrate the KC TJs by forming tricellulin-dependent TJs between KC-LC-KC. The elongated dendrites are able to reach beneath the SC and take up Ag from the extra-TJ environment without destroying barrier integrity. The original figure was published in [7].

The epidermis is composed predominantly of keratinocytes (KCs) plus a small number of Langerhans cells (LCs), melanocytes, and epidermal-resident memory T cells, as well as others. The murine epidermis also contains unique dendritic epidermal T cells, a type of *γδ*T cells. The epidermal KCs are categorized into four layers, namely, the stratum basale, stratum spinosum, stratum granulosum (SG), and stratum corneum (SC). A functional epidermal barrier depends on the existence of the SC and tight junctions (TJs) formed in the SG (Figure 1) [5, 6]. LCs can link with KCs to form TJs (Figure 1) [7, 8].

The human body contains 2–3 g of zinc (Zn), with approximately 5% of the total Zn found in the skin [9]. The concentration of Zn is higher in the epidermis than in the dermis and subcutaneous tissue, which may be due to the Zn requirement for active proliferation and differentiation of KCs [10]. Zn facilitates over 1000 enzymatic reactions and is indispensable for over 2000 transcriptional activities [11–13]. Zn finger proteins are involved in various physiological reactions [14–16]. Moreover, approximately 10% of human proteins bind to Zn [17]. Therefore, the dysregulation of epidermal Zn levels due to nutritional deficiency or genetic abnormalities of Zn transporters affects various enzymatic reactions, transcriptional activities, and Zn finger protein functions in the epidermis, leading to the disruption of skin homeostasis [18–20]. This review is aimed at highlighting the association between Zn and epidermal barrier function to understand the importance of Zn in skin immunity. We also summarize the function of Zn and Zn transporters in the epidermis and epidermal cells.

## 2. Zinc and the Epidermal Barrier

The epidermal barrier function depends largely on the presence of SC and TJs formed in the SG [4, 21]. In this respect, homeostasis of the epidermis depends on normal KC differentiation (keratinization). LCs, a type of tissue-resident macrophages, also form TJs with KCs through linkages with KCs [7]. Most of the skin is covered with stratified epithelia that have both SC and TJs. However, skin appendages such as hair follicles and sweat glands lack SC, so TJs are the sole barrier structure in the skin appendages. The sections below summarized the involvement of Zn in the formation and function of SC and TJs.

### 2.1. Stratum Corneum

The outer layer of the SC consists of a cornified layer made of flattened and denucleated KCs (or corneocytes) and a SC-specific barrier structure called the cornified envelope that replaces the KC cell membranes. Through the process of terminal differentiation, SG KCs produce two membrane-circumscribed granules, keratohyalin granules and lamellar bodies. The former contains “intracellular” components of the SC such as filaggrin (FLG), loricrin, and keratin filaments, whereas the latter contains “extracellular” components such as lipids, corneodesmosin, and kallikreins. All of these intracellular and extracellular proteins are crucial for the formation and/or function of the epidermal barrier [21].

#### 2.1.1. Zinc and Filaggrin and Its Metabolism

Among the components of keratohyalin granules, FLG has a crucial role in maintaining normal epidermal barrier function. FLG-deficient mice show an impaired SC barrier function and develop spontaneous dermatitis [22, 23]. Studies have shown that loss-of-function mutations in the FLG gene are strongly associated with the development of AD and ichthyosis vulgaris [24, 25]. These mutations were shown to range from 25 to 50% in the Northern European and Asian populations with these ailments [25, 26]. Moreover, FLG gene mutations were demonstrated as the strongest risk factor for AD in the genome-wide association studies (GWAS) [27]. These indicate the critical involvement of FLG in AD pathogenesis mediated by the disruption of the epidermal barrier.

**Figure 2:**
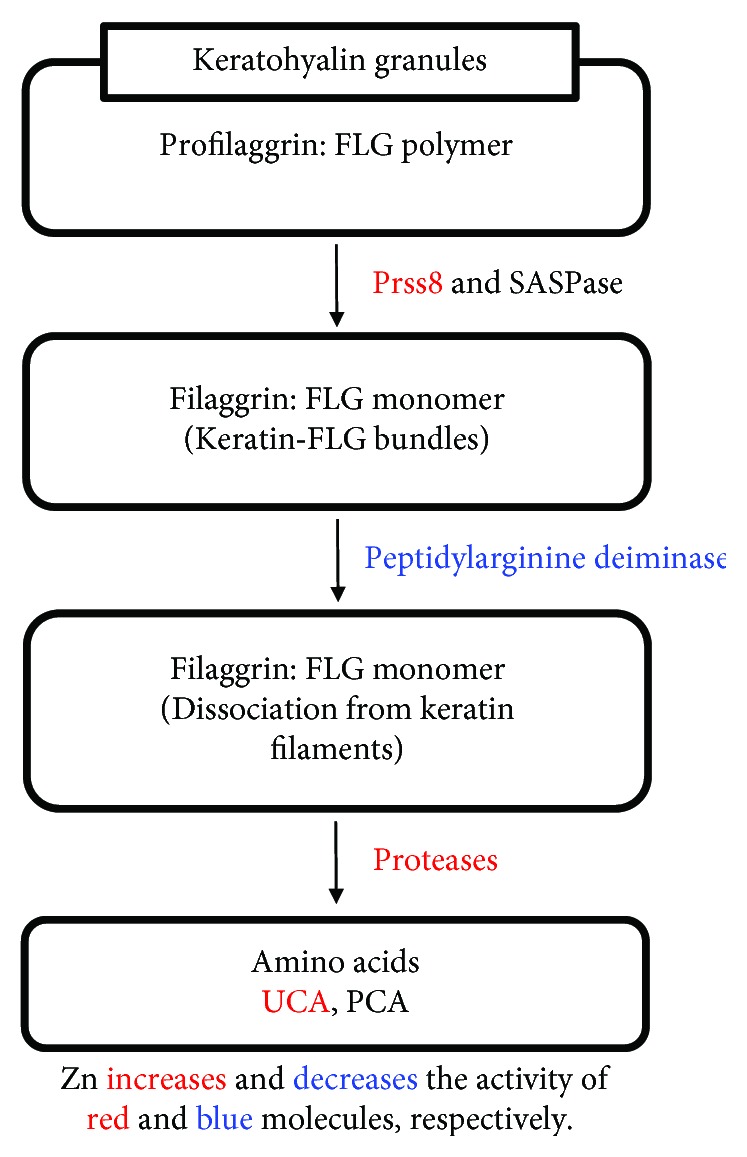
Filaggrin and its metabolism. FLG is produced in the SG as profilaggrin (FLG polymer) and is stored in keratohyalin granules. At the transition to the SC, the polymer is processed to the monomer by proteases such as Prss8 and SASPase and then binds to keratin and forms the fundamental structure of the corneocytes. At the outermost layer of the SC, FLG is citrullinated by peptidylarginine deiminase and then dissociated from keratin filaments. These dissociated FLG are degraded to free amino acids, including glutamine, arginine, and histidine. The FLG-derived histidine-rich proteins are converted into urocanic acid (UCA) and pyrrolidine carboxylic acid (PCA) by proteases. Zn facilitates FLG production by increasing the activity of Prss8. Alternatively, Zn can also suppress FLG metabolism by decreasing PAD activity. Moreover, Zn is required for histidine conversion to UCA.

FLG is produced in the SG as profilaggrin (FLG polymer) and is stored in keratohyalin granules. At the transition to the SC, the polymer is processed to the monomer by proteases such as Prss8 and SASPase [28, 29] and then binds to keratin and forms the fundamental structure of the corneocytes. At the outermost layer of the SC, FLG is citrullinated by peptidylarginine deiminase and then dissociated from keratin filaments [30]. These dissociated FLG are degraded to free amino acids, including glutamine, arginine, and histidine. The FLG-derived histidine-rich proteins are converted into urocanic acid (UCA) and pyrrolidine carboxylic acid (PCA) by proteases. UCA absorbs ultraviolet, maintains the acidic pH, and suppresses excess LC activation [31, 32]. PCA is a source of natural moisturizing factors. As a result, FLG is indispensable to the framework of SC and its metabolites are important for maintaining the epidermal barrier function (Figure 2).

Zn is involved in the regulation of FLG expression as well as its metabolism. It facilitates FLG production by increasing the activity of Prss8 [33]. Alternatively, Zn can also suppress FLG metabolism by decreasing PAD activity [34]. Moreover, Zn is required for histidine conversion to UCA [35] (Figure 2). *Propionibacterium acnes* (*P. acnes*) induce excess KC proliferation and FLG expression in the epidermis through the induction of IGF-1, which activates the IGF-1 receptor (IGF-1R) on KCs. This causes follicular plugging that is frequently observed in patients with acne vulgaris. In these cases, Zn helps to maintain homeostasis by directly suppressing the induction of IGF-1 and IGF-1R and the overexpression of FLG [36].

OVO-like proteins (OVOLs) are transcribed from ubiquitously conserved genes encoding a C2H2 zinc finger transcription factor in mammals [37]. Mutations in the OVOL1 gene, as well as the FLG gene, were demonstrated as a risk factor for AD in the GWAS [38–40]. Consistent with this finding, *Ovol1*-knockout mice show rapid disruption of the epidermal barrier [41]. OVOL1 regulates transcription in the nucleus by binding to p300, which is a member of the histone acetyltransferase (HAT) family and contains the Zn finger motif [42]. Additionally, HAT activity of p300 is negatively regulated by histone deacetylases (HDACs), which are Zn-dependent hydrolases [42]. Thus, OVOL1 expression and activity are regulated by Zn and Zn-related epigenetic enzymes. Aryl hydrocarbon receptor (AHR) is a ubiquitous ligand-activated transcription factor that is activated by both endogenous and exogenous ligands. AHR activation induces nuclear translocation of OVOL1, leading to the upregulation of FLG expression [43, 44]. Given this, it is evident that Zn and various Zn finger proteins are involved in the regulation of FLG expression.

#### 2.1.2. Zinc and Cornified Envelope, Intercellular Lipid Lamellae, and Corneodesmosome

The cornified envelope (CE) is formed of keratins enclosed within an insoluble protein shell just beneath the corneocyte cell membrane [45]. It provides a solid physical barrier and consists of a 10 nm thick layer of highly cross-linked insoluble proteins, such as involucrin, envoplakin, periplakin, loricrin, and small proline-rich protein. A 5 nm thick layer of intercellular lipid lamellae composed of ceramides, free fatty acids, and cholesterol is covalently bound to these proteins. Adhesion between corneocytes is assumed to occur by the desmosome apparatus (corneodesmosome), composed of desmosomal cadherin, armadillo proteins, and plakins. The CE, intercellular lipid lamellae, and corneodesmosome are essential for effective physical and water barrier functions in the epidermis. The role of Zn in the formation and function of these proteins has not yet been elucidated.

#### 2.1.3. Zinc and Corneocyte Desquamation

Shedding of corneocytes at the outermost layer of the SC is called desquamation and is an important step for maintaining epidermal homeostasis and preventing hyperkeratosis. This step is primarily assumed by a proteolytic cleavage of kallikrein- (KLK-) related peptidases. Out of 15 KLK proteins, human KCs express nearly all of them [46]. Among these KLKs, corneocyte desquamation is mainly conducted by KLK5, KLK7, and KLK14 [47]. The proteolytic activity is dependent on pH in the SC and is regulated by a cocktail of protease inhibitors, including lymphoepithelial Kazal-type 5 serine protease inhibitor (LEKTI) encoded by the serine protease inhibitor Kazal-type 5 (*SPINK5*) [48]. Importantly, the proteolytic activity of KLK5, KLK7, and KLK14 is impaired in the presence of Zn [49–51]. Furthermore, the expression of several KLK-related peptidases, including KLK5 and KLK7, is negatively regulated by transcriptional factor specificity protein 1 (Sp1), a C2H2-type Zn finger protein [52].

Several reports demonstrated the association between AD and Zn deficiency in humans and mice [53–55]. Consistent with this finding, the skin pH is increased in patients with AD [56], possibly due to the reduced UCA production caused by Zn deficiency (see Section 2.1.1). Additionally, Sp1 expression is decreased in the epidermis of patients with AD [52], and, as a consequence, KLK activity is often enhanced. Since KLKs process prointerleukin- (IL-) 1*α* and IL-1*β* that are abundantly stored in the corneocytes to active forms, this enhanced KLK activity leads to skin inflammation [57]. Additionally, KLK5 and KLK14 activate proteinase-activated receptor- (PAR-) 2, which is a G-protein-coupled receptor expressed on KCs, leading to the elicitation of itch common with AD [58]. Collectively, Zn suppresses excess inflammation and itching by inhibiting excess KLK activity. This action helps to maintain the epidermal barrier by suppressing IL-1-mediated FLG downregulation [57].

### 2.2. Zinc and Tight Junctions

TJs that are formed in the SG seal the intercellular spaces between SG KCs, thereby regulating the movement of water and inorganic ions via the paracellular pathway [59]. The major components of TJs are the transmembrane claudin proteins. Among the claudins expressed in the epidermis, claudin-1 is most critical for the formation and function of TJs, as confirmed in claudin-1-knockout mice, which showed severe dehydration and death soon after birth without modulating SC formation [60]. Providing further support to the importance of claudin-1, the expression is decreased in the epidermis of patients with AD [61]. Other TJ components include transmembrane proteins such as occludin, JAM-A, tricellulin, and angulins and the intracellular scaffold proteins ZO-1, ZO-2, and ZO-3.

The role of Zn in the formation of TJs has been studied in the intestinal epithelium, but less so in the epidermis. For example, it has been reported that the chelation of intracellular Zn by TPEN (*N*,*N*,*N*′,*N*′-tetrakis(2-pyridylmethyl)ethylenediamine) downregulates occludin and claudin-3, leading to TJ disruption in the intestinal epithelium [62]. It might be interesting to investigate the association between Zn deficiency in the epidermis and altered expression of TJ-associated proteins.

Zn finger E-box-binding homeobox- (ZEB-) 2 (also called SIP1) is a nuclear transcription factor that has C2H2-type Zn finger domains. Epidermal KC-specific ZEB-2 overexpression in mice showed epidermal barrier impairment along with the loss of expression of occludin and claudin-4 [63]. This suggests that the Zn finger protein, ZEB-2, negatively regulates the expression of occludin and claudin-4. The Zn endopeptidase meprin*β* is expressed in the SG KCs just below the SC. Meprin*β* is activated by KLK4 and facilitates proliferation and terminal differentiation of SG KCs, thereby contributing to the proper SC formation [64].

### 2.3. Epidermal Barrier Dysfunction and Th2 Response

Zn deficiency is known to drive Th2-type immune response and is associated with AD [65]. This suggests that Zn deficiency contributes to the disruption of the epidermal barrier through the downregulation of formation and/or function of SC and TJs. Epidermal barrier dysfunction allows external substances and stimuli to invade the epidermis and leads to the production of KC-derived Th2-inducing cytokines, including thymic stromal lymphoproteins (TSLP) and IL-33. TSLP downregulates FLG expression while IL-33 downregulates both FLG and claudin-1 expression [66–68]. Major Th2 cytokines, IL-4 and IL-13, downregulate the expression of FLG, the CE components (loricrin and involucrin), cell adhesion molecules (ZO-1), and ceramide lipids. IL-4 also inhibits the nuclear translocation of OVOL1, leading to the downregulation of FLG expression (see Section 2.1.1) [43]. IL-31, another Th2 cytokine, also downregulates FLG expression [69]. Taken together, epidermal barrier dysfunction, which is influenced by Zn deficiency, results in the disruption of skin homeostasis characterized by the Th2 immune response and vice versa.

## 3. Zinc and Langerhans Cells

LCs are antigen-presenting cells that occupy approximately 3% of the epidermis [70]. They were previously considered a subtype of dendritic cells (DCs) because of their ability to capture antigens (Ag), migrate to the draining lymph nodes (dLNs), and then present the Ag to T cells and initiate the immune response. However, recent evidence revealed that LCs originate from macrophage lineage of fetal liver progenitors and not from DC lineage [71–74]. Therefore, LCs are currently considered tissue-resident macrophages with the ability to migrate to the dLNs.

### 3.1. LCs and Tight Junctions

LCs express claudin-1 and form TJs with adjacent SG KCs [75]. As such, LCs are an important component of TJs to create an effective epidermal barrier. In steady state, LC dendrites lie beneath the TJs. Upon activation, LCs elongate their dendrites to penetrate the KC TJs by forming tricellulin-dependent TJs between KC-LC-KC. The elongated dendrites are able to reach beneath the SC and take up Ag from the extra-TJ environment without destroying barrier integrity (Figure 1) [7]. These LCs then induce Th2-type, but not Th1-type, humoral immune responses [8]. Furthermore, LCs take up KC-derived auto-Ag and present it on their MHC class II. These LCs expand polyclonal Ag-specific regulatory T cells and keep peripheral tolerance against auto-Ag [76]. Therefore, LCs assume the dual role of TJ component and surveillance agent of foreign and auto-Ag.

### 3.2. LCs and Zinc Deficiency

**Figure 3:**
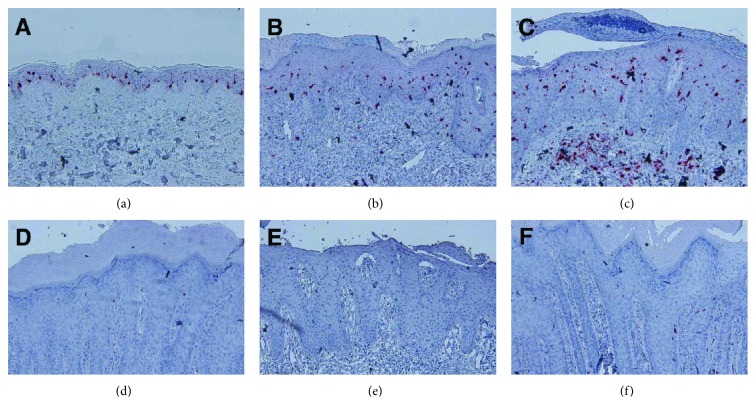
Loss of epidermal LCs in patients with AE. Immunohistochemical staining for langerin (red) in normal skin (a) and the erythematous lesions in atopic dermatitis (b), psoriasis vulgaris (c), or three AE (d–f) patients. Original magnification: 200x.

The association between Zn and LCs was revealed by analysis of skin specimen from patients with acrodermatitis enteropathica (AE; OMIM 201100) [77]. AE is an autosomal recessive disease caused by mutations in the *SLC39A4* gene that encodes ZRT/IRT-like protein 4 (ZIP4) [78]. ZIP4 is abundantly expressed in the apical side of the intestinal epithelium, thereby working as the primary gate absorbing Zn into the enterocytes. This absorbed Zn is subsequently transported to the bloodstream by Zn transporter 1 (ZnT1) [79, 80]. Therefore, ZIP4 dysfunction due to mutations results in decreased serum Zn levels. Interestingly, epidermal LCs were absent in AE skin lesions [77] (Figure 3). However, LCs were restored in the epidermis after patients were given Zn supplementation. These phenomena suggest that LCs disappeared from the epidermis when patients are deficient in Zn, but the effect can be easily reversed.

**Figure 4:**
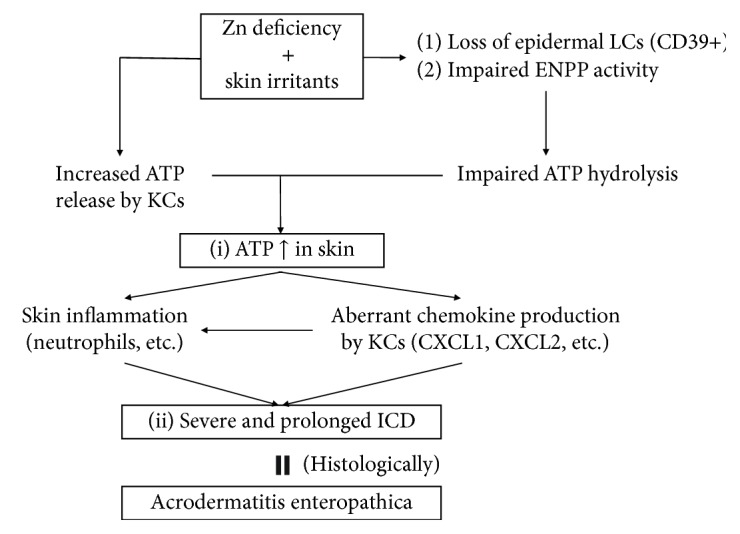
Model for the etiology of AE. (i) Skin irritants increase ATP release in the skin in Zn deficiency, due to increased ATP release by KCs, impaired ATP hydrolysis by LCs, and impaired ENPP activity. (ii) Increased ATP release induces severe and prolonged ICD via aberrant chemokine production by KCs and neutrophil-mediated skin inflammation. Histologically, cutaneous lesions in AE and ICD lesions in ZD mice demonstrate common histological features, such as subcorneal vacuolization and epidermal pallor.

The association between LC loss and the development of characteristic AE skin lesion was investigated using dietary Zn-deficient (ZD) and Zn-adequate (ZA) mice [77]. Consistent with observations in patients with AE, epidermal LCs also disappeared in ZD mice (Figure 4).

“Allergic” contact dermatitis was significantly impaired in ZD mice compared with ZA mice. On the other hand, “irritant” contact dermatitis (ICD) was significantly enhanced in ZD mice compared to ZA mice. ICD is mediated by adenosine triphosphate (ATP), which is secreted by KCs in response to environmental irritants through lytic and nonlytic mechanisms [81–84]. As expected, ATP production from croton oil-applied skin was significantly increased in ZD mice compared with ZA mice. Additionally, ATP production from Pam212 KCs (murine immortalized KCs) was significantly increased by incubation with TPEN. These data suggest that KCs in ZD mice produce more ATP than KCs in ZA mice [77].

ATP is a potent inflammation inducer. Therefore, the epidermis is equipped with a mechanism to prevent ATP-mediated inflammation in the steady state. CD39 (ectonucleoside triphosphate diphosphohydrolase-1; ENTPD-1) plays a central role in ATP hydrolysis [83]. In the epidermis, CD39 is predominantly expressed in LCs, but not in KCs [83, 85, 86]. Thus, ATP is not hydrolyzed in the epidermis of patients with AE and ZD mice, thereby eliciting ATP-mediated ICD in skin. In line with this concept, characteristic AE skin lesions develop in anogenital and periorificial areas and distal portions of the extremities, where frequent contact with external irritants including feces, urine, saliva, food, shoes, or excessive sweating is expected.

Taken together, the nature of AE skin lesions is ICD mediated by (1) increased ATP production by ZD KCs and (2) disabling of ATP hydrolysis due to the loss of CD39-expressing LCs.

As described, CD39 (ENTPD-1) potently hydrolyzes ATP. However, there are three groups of molecules that hydrolyze ATP, including ENTPDs, ectonucleotide pyrophosphatase/phosphodiesterases (ENPPs), and alkaline phosphatase (ALP) [87, 88]. The latter two molecules are Zn-dependent molecules. Among ENTPDs and ENPPs, ENTPD-1 (CD39), -2, -3, and -8 and ENPP-1, -2, and -3 aid in ATP hydrolysis. The expression of these molecules in LCs and KCs was not previously understood, with the exception of CD39. Thus, we determined that LCs strongly expressed CD39 and weakly expressed ENTPD-2 and ENPP-1, -2, and -3. Normal human epidermal KCs weakly expressed CD39, ENTPD-2 and -3, and ENPP-1 and -2. Neither LCs nor KCs expressed ENTPD-8 or ALP. Therefore, although LCs strongly express CD39, other ATP-hydrolyzing molecules are weakly expressed in both LCs and KCs. KCs occupy approximately 97% of the epidermis, whereas LCs occupy approximately 3%. Therefore, we determined the degree of contribution of LCs and KCs to ATP hydrolysis. ATP hydrolysis was impaired by approximately 80% in LC-depleted epidermal suspension compared with sham-sorted epidermal suspension. This suggests that LCs assume approximately 80% of epidermal ATP hydrolysis, whereas KCs assume the remaining 20% [89].

Recently, it was demonstrated that Zn deficiency impairs the activity of ENPP-1 and ENPP-3, as well as ALP and CD73 [90]. As described, KCs weakly express ENPP-1 and ENPP-2. This explains one underlying mechanism by which ZD KCs increase ATP production.

### 3.3. LCs and Zinc Finger Proteins

In the steady state, LCs form the firm connections with adjacent KCs by claudin-1 and E-cadherin. Additionally, EpCAM (epithelial cell adhesion molecule) in mice and its human homolog, tumor-associated calcium signal transducer 2, also participate in this cell adhesion [91]. Claudin-1 and EpCAM colocalize in LCs [92]. Upon LC activation, LCs downregulate these adhesion molecules and then migrate to the dLNs. LCs upregulate ZEB-1 and ZEB-2 (see Section 2.2) during maturation, subsequently downregulating E-cadherin expression in LCs [93, 94]. Additionally, downregulation of EpCAM in LCs impairs claudin-1 expression in LCs [92]. This suggests that Zn deficiency leads to LC retention in the epidermis. Nevertheless, the presence of LCs is reduced with Zn deficiency [77]. For LC development and survival, LC-derived, but not KC-derived, autocrine transforming growth factor beta (TGF-*β*) is crucial [95–98]. This latent secreted protein is processed and activated by *α*v*β*6 and *α*v*β*8 integrins on KCs and then is recognized by LCs [99–101]. In patients with AE and ZD mice, the epidermal expression of TGF-*β* is strongly impaired, and thus, LCs are reduced [77]. However, the association between Zn deficiency and impaired epidermal TGF-*β* expression is not fully understood.

Collectively, Zn deficiency in the epidermis results in (1) disruption of epidermal barrier function (see Section 2), (2) LC disappearance due to impaired epidermal TGF-*β* expression, (3) impaired ATP hydrolysis due to the reduced number of CD39-expressing LCs and impaired ENPP activity, and (4) elicitation of ATP-mediated skin inflammation. Because of its importance, we are currently investigating the impact of LC loss on TJ development and function using AE skin specimens and ZD mice.

## 4. Zinc and Zinc Transporters in the Epidermis

The subject of Zn transporters in the epidermis has been widely reviewed by us and others [18–20]. Thus, here, we briefly summarized what is known and added recent findings. Zn is distributed to a higher degree in the epidermis than in the dermis. Within the epidermis, Zn is distributed primarily in the stratum spinosum [102]. LCs are present in the stratum spinosum, where Zn is found, and research suggests that LCs definitively require Zn for their survival and function [77].

The fundamental functions of Zn in the KCs are proliferation and anti-inflammation. KCs treated with nontoxic concentration of Zn increased their proliferation and survival [103]. Conversely, the chelation of intracellular Zn by TPEN facilitates KC apoptosis by activating caspase-3 and DNA fragmentation [104]. Zn has been reported to suppress the production of tumor necrosis factor-*α*, inducible nitric oxide synthase, and subsequent nitric oxide in KCs [65, 105, 106]. Zn also suppresses the expression of toll-like receptor 2 on KCs [107].

The rigorous Zn^2+^ regulation is conducted by Zn transporters (ZnTs and ZIPs) and metallothioneins (MTs) [108, 109]. So far, 10 ZnTs, 14 ZIPs, and 4 MTs were identified in humans [110, 111]. ZnTs and ZIPs mediate Zn efflux and uptake, respectively. MTs are ubiquitously expressed throughout various types of cells and are predominantly distributed in the cytoplasm and to a lesser extent in the nuclei and lysosomes. MTs can bind to metal ion including Zn via a unique cysteine-rich amino acid sequence and essentially control the availability of Zn [112]. Among 24 Zn transporters (10 ZnTs and 14 ZIPs), the function of only four Zn transporters (ZnT1, ZIP2, ZIP4, and ZIP10) in the epidermis or KCs has been elucidated so far.

ZnT1 in KCs is involved in the development of epidermodysplasia verruciformis (EV; OMIM 226400), which is a rare autosomal-recessive skin disease that can lead to nonmelanoma skin cancers resulting from selective susceptibility to oncogenic human papillomaviruses (HPVs) [113]. In KCs, EVER1 and EVER2 proteins form a complex with ZnT1 primarily in the ER and to a lesser extent in the nuclear membrane and Golgi apparatus. This complex maintains Zn homeostasis, which inhibits activator protein-1 (AP-1) activation that promotes HPV replication. Patients with EV have mutations in either the *EVER1* or *EVER2* genes [114, 115]. The complex of ZnT1 and mutated EVERs increases free Zn transport in the nucleus and subsequently enhances AP-1 activity, leading to aberrant replication of EV-related oncogenic HPVs and thereby developing skin cancers.

ZIP2 is expressed on the differentiating KCs of humans and mice. Since ZIP2 knockdown in KCs decreases intracellular Zn, suppresses KC differentiation, and downregulates involucrin expression, Zn taken up by KC ZIP2 is required for proper KC differentiation and CE formation (also see Section 2.1.2) [102]. ZIP4 is expressed on the undifferentiating KCs of humans. Since ZIP4 knockdown in KCs decreases intracellular Zn, suppresses KC differentiation, downregulates the expression of FLG and involucrin, and impairs the activity of p63 that is a critical regulator of epidermal formation, Zn taken up by KC ZIP4 is required for proper KC differentiation and SC formation (also see Section 2.1) [116]. Murine ZIP10 is expressed on the epidermal progenitor cells of the outer root sheath of hair follicles. Therefore, ZIP10 depletion in keratin 14-expressing cells leads to a thin epidermis and a hypoplasia of hair follicles via downregulation of p63 activity [117]. The epidermal regulator p63 also controls the activity of ZNF750, a C2H2-type Zn finger protein. ZNF750 strongly regulates terminal epidermal differentiation. Thus, ZNF750 knockdown downregulates the expression of epidermal barrier-related proteins including FLG, loricrin, and SPINK5 [118]. In human skin, MT-1 and MT-2 are expressed in the actively proliferating KCs, such as the hair matrix, outer hair roots, and stratum basale [119]. Knockdown of both MT-1 and MT-2 in mice impairs KC proliferation [120]. MT expressions are upregulated in the hyperplastic KCs of inflamed skin lesions and skin cancers [121].

Collectively, Zn supports KC proliferation and survival. ZIP2 and ZIP4 are required for proper KC differentiation and subsequent epidermal barrier formation. ZIP10 is required for the successful epidermal formation. MTs are involved in KC proliferation.

## 5. Conclusions and Perspectives

Epidermal barrier homeostasis is the first line of defense for preventing the initiation of atopic march. SC and TJs are responsible for epidermal barrier function. Zn and Zn finger proteins regulate SC formation as well as its metabolism, while the contribution of Zn to TJ function is less well understood. However, when Zn is deficient, LCs are not present, and this important component of TJs is lacking. Therefore, Zn deficiency might lead to epidermal barrier dysfunction. Disruption of the epidermal barrier induces a Th2-type immune response by producing KC-derived Th2-promoting cytokines and T cell-derived Th2 cytokines. Meanwhile, these Th2-related cytokines impair the structure and function of the SC and TJs. In this way, epidermal barrier disruptions trigger a negative spiral of inflammation. Zn homeostasis in cells, including KCs, is maintained by ZnTs, ZIPs, and MTs. MT-mediated Zn in KCs facilitates its proliferation. On the other hand, the function of ZnTs and ZIPs in KC biology is less understood, because only the function of ZnT1, ZIP2, ZIP4, and ZIP10 has been elucidated from among 10 ZnTs and 14 ZIPs. The epidermis is composed of KCs, LCs, melanocytes, epidermal-resident memory T cells (TRMs), and others. The murine epidermis, but not the human epidermis, also contains dendritic epidermal T cells (DETCs), a type of *γδ*T cells. The role of Zn and Zn transporters in LCs, melanocytes, TRMs, and DETCs has not been analyzed to date. Additional research must be conducted to thoroughly understand the role of Zn and Zn transporters in maintaining healthy skin.
